# “How to save a marriage”: treatment of REM sleep behavior disorder (RBD) with acetyl-leucine in a patient with Parkinson’s disease

**DOI:** 10.1007/s00415-025-13195-w

**Published:** 2025-06-17

**Authors:** Wolfgang H. Oertel, Martin T. Henrich, Filip Bergquist, Annette Janzen, Fanni F. Geibl, Michael Strupp

**Affiliations:** 1 Department of Neurology, Philipps University Marburg, Baldingerstrasse 1, Marburg, Germany; 2 Institute of Neurogenomics, Helmholtz Institute for Medicine and Environment, Munich, Germany; 3 Department of Psychiatry and Psychotherapy, Philipps University Marburg, Marburg, Germany; 4 Institute of Neuroscience and Physiology, University of Gothenburg, Gothenburg, Sweden; 5 Department of Neurology, Sahlgrenska University Hospital, Gothenburg, Sweden; 6 Department of Neurology, LMU University Hospital, LMU Munich, Munich, Germany

Dear Sirs,

Isolated REM sleep behavior disorder (iRBD) is a specific prodrome of the α-synucleinopathy Parkinson’s disease (PD) [1]. The phenotype RBD also occurs in up to 60% of patients with manifest PD [2]. Direct evidence on therapeutic options for the phenotype RBD in PD is lacking. Therapy of the RBD phenotype in PD is instead based on consented therapeutic recommendations for the symptomatic treatment of subjects with isolated RBD. Respective guidelines are based on limited evidence derived from clinical studies and trials mainly with clonazepam and melatonin [3].

The use of acetyl-leucine in a patient suffering of Parkinson’s disease in combination with the phenotype RBD was based on an accidental finding in the past: In 2020, we treated a PD patient, who also presented both with Restless Legs Syndrome (RLS) and RBD, with the modified amino-acid acetyl-DL-leucine (AL; 5 g/day orally) for his RLS symptoms [4]. 5 weeks after initiating AL treatment, not only the RLS symptoms were reduced, but the patient reported a marked improvement in the two key clinical features of the RBD phenotype—the disappearance of aggressive dreams and a considerable decrease of dream enactment. Subsequently, a similar substantial long-term improvement of RBD symptoms was reported by us in two subjects with isolated RBD with AL [5]. Based on these observations, we tested the effect of AL in a patient with manifest PD, who also suffered from marked RBD, over a period of 2 years.

In 05/2022, we saw a 67-year-old Swedish male citizen (see Supplementary Material for ethical considerations), who received the clinical diagnosis of iRBD in 2016 based on the description of symptoms by the patient and his wife. He subsequently started himself on treatment with melatonin (5 mg immediate release). In 2019, the patient reported mild slurring of speech and difficulties with fine motor tasks. On examination, a reduced facial expression, mild akinesia in the right arm and a reduced right arm swing on walking were observed. An MRI and laboratory tests were normal (Supplementary Material Table S1). The diagnosis of PD was made. In the following years, his PD motor symptoms were controlled with a therapy of 3 × 100 mg levodopa/25 mg benserazide (later levodopa/carbidopa) plus rasagiline 1 mg/day. In contrast, the severity and frequency of his RBD symptoms substantially increased and only partially responded to treatment with melatonin (5–15 mg/day immediate release, later plus 2 mg controlled-release). As a therapeutic alternative, the treating neurologist (FB) offered and prescribed him a therapy with clonazepam. The patient, however, refused to take this drug, as he was afraid of becoming addicted to benzodiazepines and in addition feared the effect of clonazepam on his ability to drive a car. With time, the severity and frequency of the patient’s aggressive dreams and their enactment in form of violent large-amplitude movements of his extremities increased. Therefore, the couple placed a large pillow between them, to prevent the movements from injuring the wife and allow the couple to share the bedroom (Fig. 1a).

**Fig. 1:**
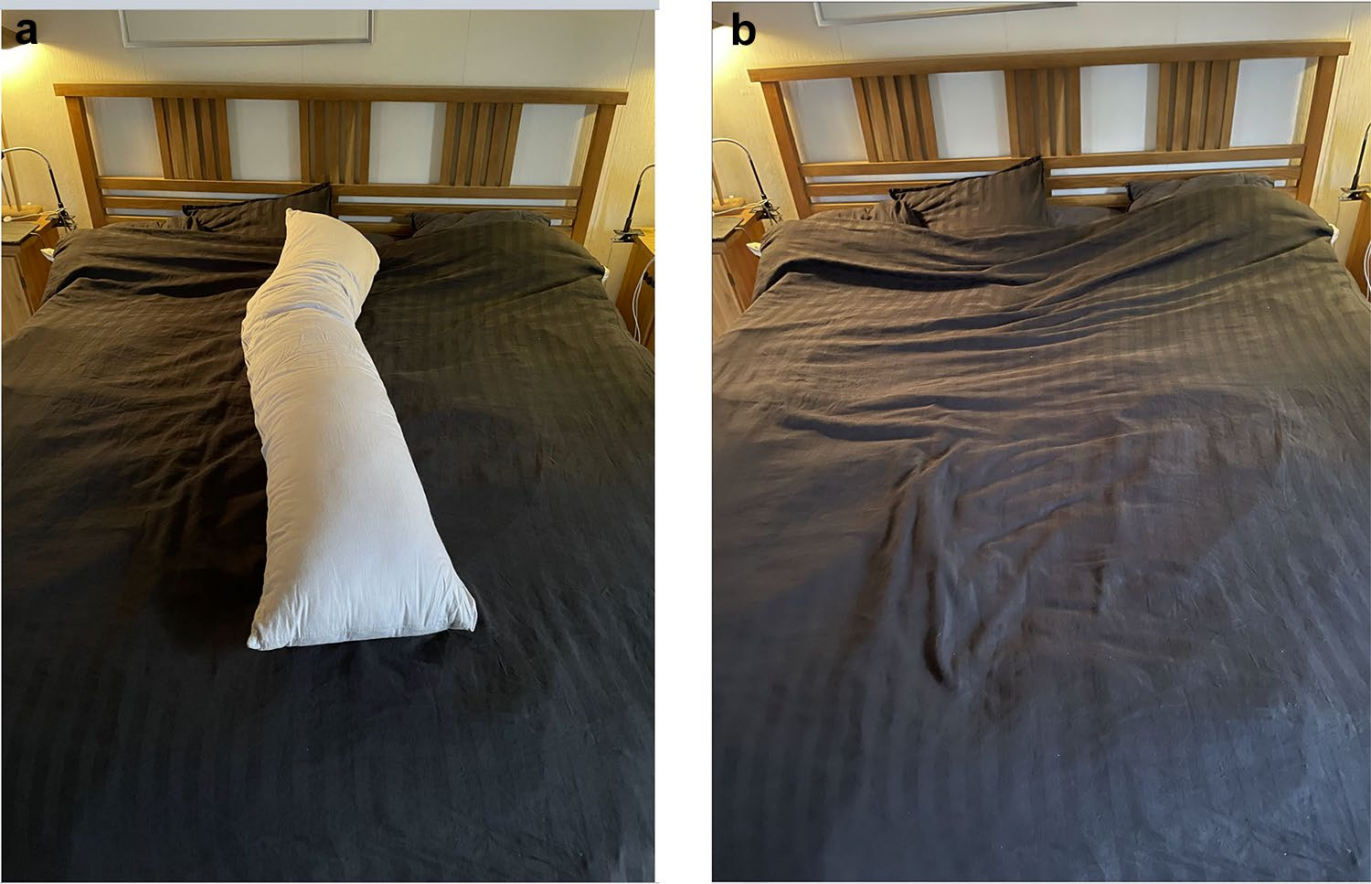
Photographs of the bedroom before (**a**) and under therapy (**b**) with acetyl-leucine

Acetyl-DL-leucine is the racemate of acetyl-d-leucine and the bioactive enantiomer acetyl-l-leucine. Acetyl-dl-leucine is approved and marketed under the tradename Tanganil^R^ for the indication vertigo in France since 1960. The drug is considered safe and well tolerated. Acetyl-l-leucine has been approved in autumn 2024 by the Food and Drug Administration in the USA for the indication Niemann–Pick disease type C under the trade name Aqneursa^R^ and is marketed by the company IntraBio. For reasons of availability in the year 2022, we employed the racemate acetyl-DL-leucine in this observational case study.

In the first week of 06/2022, the patient agreed to take AL under the rules of “an individual case of OFF label use”. In order to minimize bias, the treating Swedish neurologist (FB) received only the information that AL might have a beneficial effect on RBD symptoms. Neither the neurologist, nor the patient nor his spouse obtained any information about the latency to onset of the effect, the type or degree of efficacy of AL on the different aspects of RBD.

During the second washout, the patient underwent additional investigations to substantiate the diagnosis of PD and RBD. These investigations included a standardized clinical assessment with the MDS-UPDRS, a detailed neuropsychological examination, a video-assisted polysomnography (vPSG), dopamine transporter (DAT) ligand-binding imaging (DAT-SPECT) and an olfactory function test with the University of Pennsylvania Smell Identification test (UPSIT) (for methodological details, see Supplementary Material). In addition, we captured the overall subjective assessment of the effect of the AL therapy on the RBD phenotype by asking the patient and spouse separately to answer a list of questions related to the frequency and severity of his RBD phenotype (Table 1).

**Table 1:** Subjective assessment of the effect of acetyl-leucine on RBD phenotype by patient and spouse

	Patient	Spouse
Did you have a severe RBD phenotype with violent movements and aggressive dreams before the therapy with AL?	Yes—severely	His RBD frequently woke me and I had to wake him up in order to avoid being punched. We introduced a long pillow between us when we sleep—for my protection
Are these severe stages of RBD present under AL therapy?	Much less	It is much less frequent and much less severe. I sleep better and he does not wake me most nights. We no longer need the pillow
Is the frequency the same, did the frequency increase or decrease?	Markedly decreased	There are likely small movements and vocalizations more often than I can monitor, since they do not wake me up
Which stages of severity do you or your partner notice under AL treatment?	Using the scoring system, typically I would realize I had been dreaming but unsure of severity upon wakening, for any score above 2	I rate 1 if I notice him talking or moving a little at least once in the night. I wake him if I score 2 or above. At score 2, it is because I want to sleep. At score 3, I am afraid that he could punch me and at score 4, that he could harm himself (e.g., by hitting his arm on the bedside table or sleep-walking). Under AL therapy, I have rarely scored a 4 and occasionally a score 3
Is the content of your dreams aggressive? Please describe the quality of your dream content	Not always. Often in disagreement with someone but only occasionally afraid. A common theme is trying to gain social status. Usually, I am not the aggressor. Another theme is fear of heights	When he is having a nightmare, he can speak/shout full sentences, only in his mother tongue, Swedish. These usually coincide with movement. The more severe the movement, the louder he shouts. Sometimes he laughs and vocalizes as if he was awake. If I wake him, the amount of vocalization/movement coincides with his description of a pleasant dream or a nightmare
Did you notice any particular trigger to induce RBD phenotype?	No	We explored the idea, suggested by his clinicians, that it could be worse if he takes alcohol, but I do not think so. He never drinks more than 1–2 glasses of wine per night at weekends and his scores were not obviously worse on the occasional days he drank
Did the severity and frequency of the RBD symptoms change in the last 6 months?	Do not believe so	His spoken language during RBD episodes is easier to understand
Did you change your previous medication for RBD?	No	No
Any other comment?	The fact that I plan to continue to take the drug speaks for itself	I want him to continue on the drug, reflecting on how he was before starting to take it
Adverse effects	None	

The video-assisted polysomnography (vPSG) [6] confirmed the diagnosis of RBD. Dopamine transporter single-photon emission computerized tomography (DAT-SPECT) was pathological and the olfactory function tested with the University of Pennsylvania Smell Identification Test (UPSIT) revealed hyposmia (8 of 40 (normal range) points) (for further results, see Supplementary Material Table S1).

For the key clinical patient-related outcome measure, the “7-day-RBD_severity sum-score” (7d-RBD_SS) was employed which is a modified version of a published RBD diary (modified from Kunz et al. 2021) [7]. The original version of the RBD diary rates the mildest RBD phenotype “talking and/or mild movements/jerks” as a “0”, lacking a gradation of no RBD symptoms. In contrast, the new modified version defines this level of mild severity as “1”, whereas the score “0” describes “no RBD symptoms”. Thus, this modified rating scale offers 5 levels of severity (0 = no RBD symptoms; 1 = talking or mild movements/jerks; 2 = shouting and/or complex non-aggressive movements; 3 = movements violent and large enough with a risk to injure the patient her-/himself or the bed partner; 4 = movements leading to fall out of bed) allowing to differentiate the milder forms of the RBD phenotype in two levels of severity. It should also improve the chance to capture a therapy-related change in symptom severity.

The effect of AL on the severity and frequency of the RBD phenotype was recorded by the patient and his spouse on a daily basis. Seven subsequent daily scores were summed up to obtain the “7-day-RBD_severity sum-score” (7d-RBD_SS—for details, see Supplementary Material).

The clinical outcome measure (7d-RBD_SS) was 14 at baseline. After 4 weeks under AL-therapy, it dropped below 6. At this time, the patient noted a dramatic improvement in the severity of the aggressive dream content and of violent movements. In the following 4 weeks, the 7d-RBD_SS further fell to 1 (Fig. 2). Aggressive dream content disappeared entirely. During the following 6 weeks the 7d-RBD_SS varied between 1 and 4. At this time, the couple removed the pillow on their bed (Fig. 1b). At week 16, a preplanned AL washout was performed. The beneficial effect of AL was maintained until week 22. In week 23, i.e., after 7 weeks of AL withdrawal, RBD symptoms reappeared and increased from an 7d-RBD_SS of 2 to an 7d-RBD_SS of 11 in week 23 and subsequently showed a marked rebound to a value of 18 during weeks 25 to 26. AL therapy was restarted in week 23 and with a latency of 4 weeks the 7d-RBD_SS decreased to 2 in week 27 and at week 30 scored 4.

**Fig. 2:**
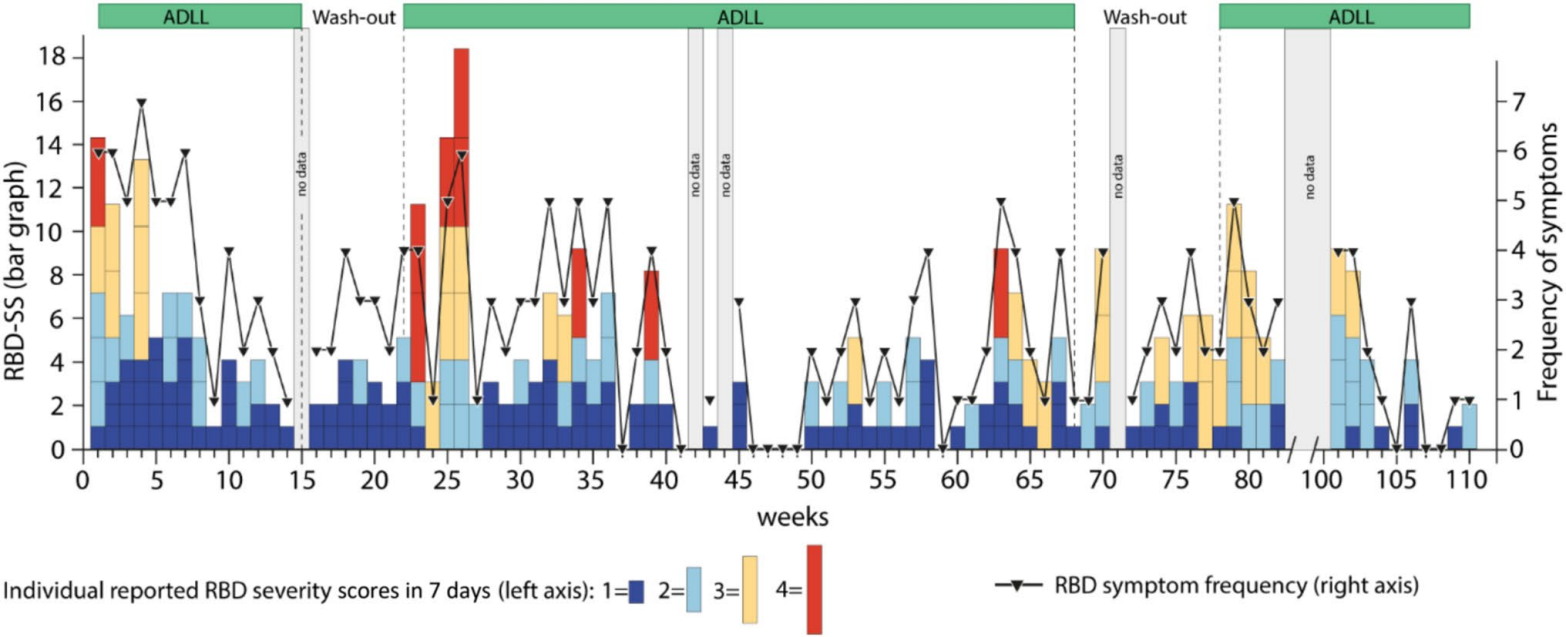
7-day-RBD_severity sum-score (7d-RBD_SS) during acetyl-leucine therapy and washout phases. After 4 weeks of AL therapy, severity score 3 (light orange) and score 4 (red) disappeared. Maximal effect on the 7d-RBD_SS reflected by a low severity score value of 1 per week (dark blue) was recorded by patient and spouse in week 9. Wash-out of AL was carried out in week 16. Note the long-lasting beneficial effect of AL on the RBD-severity score until week 22 and the marked rebound in RBD symptoms (reappearance of score value 4 (red)). These severe RBD scores were controlled by reintroducing the AL therapy—with a latency of 5 weeks. Following the first AL withdrawal, overall, during the subsequent observational period of 17 months under AL therapy and independent of the second washout period, only 3 short episodes with a marked RBD-severity score 4 (red) were reported

This effect of AL on 7d-RBD_SS was—apart from rare reoccurrence of mild to marked RBD symptoms (week 34, 39 and weeks 63 to 64; 7-RBD_SS: ≤ 9)—maintained for the next 38 weeks (up to week 68). A similar pattern in the time course of the 7d-RBD_SS was obtained during (weeks 69–78) and after the second withdrawal period (Fig. 2). The marked improvement of the RBD phenotype (the 7d-RBD_SS varied between 0 and 4) under AL therapy was maintained in the following months up to the end of the 2-year observational period. Overall, the couple rated the effect of AL on the severity and frequency of RBD as good to very good (Table 1). AL was well tolerated.

This article describes a therapeutic effect of the modified amino-acid acetyl-leucine (AL) on RBD, a frequent non-motor symptom, in Parkinson’s disease. Over a 2-year period, AL treatment provided a sustained marked beneficial effect on the severe RBD phenotype of the participating PD patient. The “latency to onset of effect” was 4 weeks. The full impact on the severity and frequency of the RBD phenotype including disappearance of aggressive dream contents evolved at 9 weeks. After a withdrawal of AL, its effect was maintained for 7 weeks followed by a strong rebound. Reintroduction of AL therapy again provided control of RBD symptoms. This pattern was reproduced by a second AL withdrawal 1 year later. These observations make it unlikely that the effect of AL is entirely due to a placebo effect. The patient and spouse observed, despite the continuous intake of a stable AL therapy, that phases of high AL efficiency with nearly RBD-phenotype-free nights alternated with phases of mostly mild to moderate, rarely marked RBD severity. The reason for this fluctuation is not clear, as the couple failed to identify triggers for inducing or aggravating the RBD phenotype [8] .

AL has been reported to have multiple mechanisms of action. It improved neurological symptoms in cerebellar diseases [9] and in particular in lysosomal storage disorders [10, 11], namely in Niemann–Pick disease Type C [12–14] and Tay–Sachs disease [11]. In vitro experiments showed that it normalized membrane potential and neuronal excitability [15], improved lysosomal function and also enhanced the energy homeostasis of neurons [16] .

Overall, AL had a beneficial effect on the RBD phenotype (for discussion, see Supplementary Material) in the reported PD patient. This effect was sustained over a washout period of about 7 weeks. Our observation suggests that AL leads to a modification in the function of structures responsible for the phenotype RBD—for example in the circuitry of the locus coeruleus/subcoeruleus complex or the medial ventral medulla [17] .

This case report has several limitations. We report the effect of AL in a single PD patient. Our study was open label. Due to the previously observed delayed onset of effect in iRBD [5], we did not perform a drug-free baseline assessment or a run-in period with a placebo, but instead two washouts. The patient-related clinical outcome measure relied on self-reporting of the 7d-RBD_SS by patient and spouse (for a detailed discussion of the limitations, see Supplementary Material).

We conclude that AL appears to markedly reduce the phenotype RBD in PD for up to 24 months. Based on these findings, a randomized, placebo-controlled trial with the bioactive enantiomer acetyl-L-leucine [18, 19] in PD patients with RBD should be considered.

## Supplementary Information

Below is the link to the electronic supplementary material.

Supplementary file1 (DOCX 39 KB)

## Data Availability

Single research data points are presented in Figure 2 in the Letter to the Editors. Raw source data are provided on reasonable request by the corresponding author. The data can be shared publicly as the participant consented to the sharing of data as per European Union’s General Data Protection Regulation (EU GDPR) and the corresponding German and Swedish privacy laws (see also the respective statement in the Supplementary Material).
