# Supplementary material for: “How to save a marriage”: treatment of REM sleep behavior disorder (RBD) with acetyl-leucine in a patient with Parkinson’s disease
Source: J Neurol. 2025 Jun 17;272(7):466. doi: 10.1007/s00415-025-13195-w (PMC12174258; doi:10.1007/s00415-025-13195-w)
Supplement: Supplementary file 1 — Supplementary file1 (DOCX 39 KB) [file 415_2025_13195_MOESM1_ESM.docx]

**Letter to the Editors – Journal of Neurology – JOON-D-25-00888**

**Supplementary material to**

**“How to save a marriage”: Treatment of REM Sleep Behavior Disorder (RBD) with acetyl-leucine in a patient with Parkinson’s disease**

Wolfgang H. Oertel^1,2§^, Martin T. Henrich^1,3§^, Filip Bergquist^4,5^, Annette Janzen^1^, Fanni F. Geibl^1,3*^, Michael Strupp^6*^

**Acetyl-leucine therapy - individual case of OFF-label use - patient consent**

Acetyl-leucine (Tanganil^TM^) is approved in France for the treatment of vertigo. The only relative contraindication is gluten-sensitivity. The patient provided written informed consent to additionally receive - under “an individual case of off-label use” rules - a dosage of 5 g/d of AL (Tanganil®) (1 g in the morning, 1 g at noon, 3 g in the evening – a dosage previously used for other indications^1-3^) for up to 2 years. The patient consent form was also signed by the informing physician (WHO) and the document was kept in a safe with access only to WHO.

**Ethical Considerations**

The patient was treated in accordance with the Helsinki declaration, article 37.

Initially treatment with acetyl-leucine was administered under the German legislation for “individual case of OFF-label use/compassionate use” as this drug is approved for marketing only in France for a different indication (vertigo). The patient is, however, a Swedish resident. Acetyl-DL-leucine (AL) is - likewise - not approved for marketing for any indication in Sweden. As treatment was known and again found to be well tolerated and perceived efficacious, for the patient an individual license for use of acetyl-DL-leucine was sought by the treating neurologist in Sweden (FB). This individual license secured the supply of the compound. The individual license was approved (as per 3NOV2022) to receive AL (Tanganil) in the OFF-label indication RBD by the Swedish Medical Products Agency. This license is renewed on a yearly basis as long as there is no safety concern or until the drug is registered (for any indication) in Sweden.

The review board of the ethics committee at the University of Marburg has ruled that the administration of a compound as “individual case of OFF--label use” is exempt from ethical vetting. The Swedish Ethics Review Appeal Board has ruled that clinical case reports describing individual accounts of diagnostics and treatment should not be subjected to ethical vetting in Sweden. Thus, the need for approval of the protocol and the “Individual case of OFF-label use” was waived by both institutions.

**Clinical outcome parameter: weekly RBD severity sum score (7d-RBD_SS) for severity and frequency of RBD-symptoms**

The effect of AL on the daily severity and frequency of the RBD-phenotype was recorded by the patient and his spouse using a modified version of a published RBD diary (modified from Kunz et al. 2021^4^). This *modified* diary has a ranking scale for

(a) severity (score range 0-4; higher values indicate higher severity) with 0 = no RBD symptoms, 1 = speaking or mild jerks, 2 = shouting or complex, non-aggressive movements, 3 = aggressive movements with the risk to injure her-/himself or the partner, 4 = movements, which are so violent, that the person falls out of bed and

(b) frequency (score ranges from 0 to 3, 0 = no RBD symptoms, 1 = 1 to 2-nights per week RBD symptoms, 2 = 3 to 5-nights per week RBD symptoms to 3 = 6 to 7 (daily) RBD-symptoms). In variance to the frequency scale of the original diary,^4^ we present the frequency with a range of 0 – 7 (0, no RBD symptoms, 1 = RBD symptoms one night per week; 7 = daily RBD symptoms).

The filled out anonymized form was sent every Sunday by the patient to the principal investigator. Seven subsequent daily RBD-severity scores were summed up to a “7days-RBD-severity sum-score (“7d-RBD_SS)" (range of 0 to 28). This value was used as the clinical patient-related outcome measure. The “7d-RBD-SS“ was plotted into a diagram over the observational period of 24 months (see **Figure 2** – Letter to the Editor).

**Additional investigations**

***Video-assisted polysomnography (vPSG)***

Between 2014 and 09/2023, the diagnosis of RBD was made and accepted based on the description of the patient and his wife and amateur videos made at night by the wife of the patient. In order to fulfill the criteria for a definite diagnosis of RBD, an in-patient video-assisted polysomnography (vPSG) was performed in November 2023. To prepare for this diagnostic step, the patient stopped AL-therapy on September 24^th^ 2023 and it was waited until patient and spouse reported the reappearance of RBD symptoms – although to a lesser degree of severity and frequency than observed in spring-summer 2022, i.e., in the period before treatment with AL had been initiated. vPSG was performed according to AASM criteria^5^ and evaluated blindly by the staff of the Sleep Laboratory of the Department of Internal Medicine in Gothenborg, Sweden. The diagnosis of RBD was confirmed.

In response to the reviewer’s comment, we sought to arrange a second polysomnography in a certified sleep laboratory at the academic medical center at the University of Gothenborg, as well as at several accredited sleep centers in the greater Gothenborg area. Unexpectedly, we were informed that the earliest available appointment would not be in the year 2025.

We agree that a second polysomnography would provide additional objective data and complement the current analysis regarding the effect of Acetyl-DL-Leucine on the RBD phenotype. However, we believe it would not alter the main conclusion of our report – that both the patient and his spouse perceived a marked reduction in the severity and frequency of RBD symptoms following treatment with acetyl-DL_leucine.

It is important to note that while polysomnography offers a standardized and objective snapshot of sleep behavior over one or two nights in a laboratory setting, it represents an artificial environment for sleeping. In contrast, the RBD diary allows virtually continuous (night by night), albeit subjective assessment by the couple under real life conditions. Therefore, in summary, we do not consider a second polysomnography essential to prove the efficacy of Acetyl-DL-Leucine in RBD in this Parkinson patient.

***Dopamine-transporter (DAT) ligand-binding imaging (DAT-SPECT)***

The DAT-SPECT was performed in 11/2023 at the Department of Nuclear Medicine, Sahlgrenska University Hospital, Gothenburg, Sweden using the GE Health Care system camera Tandem 870 CZT. Signals were analyzed using DaTQUANT software according to clinical routine procedures. The numeric values of the striato-occipital binding ratios are listed in Table S1.

***Olfactory function test with the University of Pennsylvania Smell Identification test (UPSIT)***

In 2019 on the first consultation with his neurologist (FB), the patient was asked about his sense of smell and denied then any impairment of subjective olfactory function. In autumn 2020, he lost his olfaction due to COVID-19 infection. He reported in September 2021 that olfactory function had partially recovered. The first formal testing of olfactory function was performed at home by the patient with the smell identification test “UPSIT” at the end of October 2023, i.e., about 4 weeks after he had withdrawn AL for the second time and shortly before the video-assisted polysomnography was performed (see above).

***Neuropsychological testing***

Montreal Cognitive Assessment (MoCA) and MiniMental State Examination (MMSE) testing were not carried out. Instead, a comprehensive neuropsychological assessment was performed in August 2022 and again in December 2023. The test battery consisted of Rey Auditory Verbal Learning Test (vs. 1) , revised Brief Visuospatial Memory Test (vs. 1); Rey Complex Figure Test; number repetition in Wechsler Memory Scale III; Wechsler Adult Intelligence scale IV; Trail Making test condition 2 and 4; Color-Word interference test from the Delis Kaplan Executive function test; Boston Naming test; the F-A-S word fluency test, semantic fluency test; line orientation vs. V.

The evaluation summary of the first assessment showed a normal or above normal performance in most evaluated domains, but an uneven learning profile and increased sensitivity to interference in verbal memory tests. Psychomotor tempo, visual scanning and working memory were reduced and reduced speed was observed in tests requiring parallel processing.

Due to the later deficits, rivastigmine 9.5 mg/d was introduced at the end of August 2022 – 3 weeks before the first washout of AL. In the repeated test battery in September 2023, the performance had decreased somewhat in visual scanning and visual attention, but was otherwise stable.

**Table S1 Clinical data and results of additional tests**

| ***Clinical history and therapy*** | |
| --- | --- |
| Age at baseline | 67 years |
| Beginning of symptoms of RBD according to statement and spouse | 2014 |
| Clinical diagnosis of RBD | 2016 |
| vPSG-confirmed diagnosis of RBD | 2023 |
| Therapy of RBD since 2016 | Melatonin 5-15 mg/d immediate release, since 2022 in addition 2 mg controlled release |
| Diagnosis of PD (clinical) | 2019 |
| Therapy for PD 2019 – 2022 | 3 x 100 mg levodopa/ 25 mg benserazide plus 1 mg rasagiline |
| Therapy for PD since 2023 | 4 x 100 mg levodopa/ 25 mg carbidopa plus 1 mg rasagiline |
| Therapy for mild cognitive deficits since the end of August 2022 | rivastigmine 9.5 mg/d transdermal patch |
| Hoehn and Yahr stage in ON 2/2022 | 2.5 |
| ***Laboratory tests and imaging*** | |
| **CSF - related to neurodegeneration**   - neurofilament light chain - amyloid-Beta42/40 - GFAP - total tau/phospho-tau | all normal |
| **routine CSF** – cell counts, protein level | normal |
| **MRI of the CNS** | normal |
| **DAT-SPECT** (2023) | striatal to occipital binding ratios:  striatum right +1.18 (-51% deviation)  striatum left +1.11 (-54% deviation)  putamen right +1.07 (-54% deviation)  putamen left +1.01 (-56% deviation)  caudate n. right +1.43 (-45% deviation)  caudate n. left +1.33 (-49% deviation) |
| **Olfactory function (UPSIT)** | 8/40 – hyposmia |

**Neuropsychological testing** see Text

***Effect of AL on the Parkinson symptoms ?***

In the years 2020 to 2022, before the therapy with AL was initiated, the following scores were available: MDS-UPDRS, CISI-PD, PRO-PD and PDQ 8 – all in ON.

Therapy with AL was started in June 2022.

MDS-UPDRS, CISI-PD, PRO-PD and PDQ8 were repeated in August 2024 – all in ON.

**MDS-UPDRS:** range - the higher the score, the more severe the symptoms

2021-09-01: part I 8, part II 4, part III 28, part IV 3, total 43

AL since 06/2022:

2024-08 : part I 14, part II 6; part III 31, part IV 3, total 54

**Total Clinical Impression of Severity Index for Parkinson’s disease (CISI-PD) scores:**

range - the higher the score, the more severe the symptoms

2020-02-28: 2

2020-11-02: 2

2021-09-01: 5

2022-10-28: 7

AL since 06/2022:

2024-08 : 7

**Patient reported outcome measure for Parkinson Disease (PRO-PD) scores**:

range - the higher the score, the more severe the symptoms

2020-01-01: 338 very good (301-600)

2020-11-02: 245 excellent (1-300)

2021-09-01: 241 excellent (1-300)

AL since 06/2022

2024-08 : 571 very good (301-600)

**PD-Quality of Life (PDQ) 8:** range - the higher the score, the worse the quality of life

2020-01-01: 2 (6.3%)

2020-11-02: 2 (6.3%)

AL since 06/2022:

2024-08 3 (9.4 %)

During the AL-treatment time of 24 months no systematic clinical rating in respect to his Parkinson’s disease symptoms was performed. When questioned about his motor and cognitive conditions, the patient and also the observing spouse did not notice any change in the first 12 months. Under the combination therapy with levodopa/carbidopa and the MAO-B-inhibitor rasagiline the morning akinesia was mild and unchanged in its character as were the rare wearing-off periods. However, during the course of the observational period of months 13-24, the patient reported some shortening of the effect of the individual levodopa dose and requested to increase his symptomatic therapy. Accordingly, the levodopa dose was slightly raised from 3 times to 4 times 100 mg levodopa /25 mg carbidopa/daily by the treating neurologist.

It must be mentioned that the patient started on his own exenatide in January 2020 and continued until it was no longer obtainable (early 2023). He later sourced Ozempic for a while too, but stopped this therapy during the second half of 2023.

**Limitations**

***The primary outcome measure is a patient-centered subjective RBD phenotype severity rating – using a 7-days-RBD severity sum-score (7d-RBD_SS)***

The gold standard for an objective assessment of the RBD phenotype is video-assisted polysomnography (vPSG). This procedure requires an in-patient overnight recording for one to two nights in a certified sleep laboratory. Thus, it is costly and time-consuming and not available on a daily basis. We therefore did not rely on few repeated vPSG, but employed a recently published RBD diary^1^ and modified it slightly (see section above). The instrument allows “continuous” daily scoring of the severity of the RBD phenotype by patient and spouse. In addition, the daily rating of the RBD-phenotype allows us to obtain additional information about the type of dream content, especially in relation to an aggressive nature. Reduction or disappearance of aggressive dream content can best be assessed by the patient and indirectly by the spouse (**Table 1** –Letter to the Editor). Overall, the early morning scoring of the events in the previous night reflects whether a compound provides a benefit for the patient and also for the spouse and is thus a clinically relevant outcome measure.

Nevertheless, we are aware of the limitations of the 7d-RBD-SS as the only outcome measure:

First, the employed RBD-diary has not been validated in a multicenter study.^4^

Second, the recording of the RBD-severity score is subjective and reflects the impression of the patient and spouse.

Third, the content of the dreams and especially the aggressive nature of dreams was not systematically assessed.

Fourth, if the RBD event was not strong enough to wake up the spouse, mild RBD events (severity level 1, may be even severity level 2) were missed. And we are aware that the quality of sleep of the spouse substantially contributes to whether the spouse notices RBD symptoms of the patient. Thus, the documentation of the RBD-severity and frequency most likely underrepresents the frequency and severity of the mild to moderate RBD phenotype established before and under AL-therapy. From the perspective of the patient and spouse, however, this is not relevant, as the disturbing feature of RBD in general is the disruption of sleep by severe RBD symptoms (shouting, hitting) and the spouse’s fear of being injured by the patient’s violent dream enactment.

Fifth, if the spouse is not present at night or sleeps in a different room or if the patient does not have a partner, underscoring of the RBD-severity and frequency by the patient alone is highly likely.

To partially overcome the limitations, we asked the patient and spouse to answer a list of questions related to the frequency and severity of his RBD phenotype (**Table 1**– Letter to the Editor).

***Unblinded study***

The participating patient was not blinded. Two of the authors (WHO, MS) knew from another PD patient with additional RBD phenotype (mentioned in ^6^) that an effect on the RBD phenotype might be expected after about 5 weeks. Additional previous experience with AL in RBD relates to two subjects with isolated RBD ^6^, in whom the onset of AL-effect was observed after the third week. Thus, neither the principal investigator nor the treating neurologist were certain about the degree of effect to be expected and at what time-point an initial effect of AL might be predictably observed.

The patient was informed that AL may have a beneficial effect on his RBD, but he did not receive any information on whether an effect would be related to the dream content or the dream enactment. Likewise, the patient and his wife were not informed that the effect of AL in another PD patient with RBD had been observed with a delay of 5 weeks. Thus, from a pragmatic – but not from a formal – point of view the patient was somehow blind.

***Lack of a treatment-free baseline***

Based on the statement of one PD patient^6^ that the latency to onset of the AL effect is 5 weeks, we assumed that we would have a period of 5 weeks to establish a baseline score of the RBD severity and did not perform a run-in period without treatment.

This is a limitation of the study, as we do not have a true baseline score without AL-treatment. Nevertheless, the data show that there is a marked difference between the severity and frequency of RBD-events in the first 5 weeks and in all other weeks of the 24-month observation period (**Figure 1** - Letter to the Editor).

***Lack of studies on whether AL provides a disease modifying effect in manifest PD***

We did not investigate whether AL might have provided any modifying effect in manifest PD. This aspect needs further investigations, preferentially in subjects in the early manifest PD stage - combined with or without RBD.

***No test for establishing an alpha-synucleinopathy***

There was no doubt that the patient had presented with RBD in the prodromal phase of PD. He now suffers from a levodopa-responsive Parkinson’s disease of “late-onset” combined with hyposmia and has a reduced DAT-SPECT striatal binding ratio. We did not perform a skin biopsy to look for aggregated phospho-alpha-synuclein in dermal nerve fibers^7^ nor did we assay the total content of alpha-synuclein in serum neuronal extracellular vesicles (neuronally derived exosomes).^8^ From the literature it is known that subjects with isolated RBD with hyposmia have a very high chance to present phospho-alpha-synuclein aggregates in skin biopsy and to possess an increased alpha-synuclein content in neuronally derived serum exosomes.^7,8^ In summary, it is likely that the underlying pathology of the Parkinson’s disease described in this case report is an alpha-synucleinopathy.

**Data Availability - consent to publish research data**

The patient provided written consent for the publication of case data. He also agreed to provide all data to the scientific community without any restrictions in respect to time and scope of the data.

All measures have been taken to protect the patient’s identity. Data can be shared publicly as the participant did explicitly consent to the sharing of his data as per the European Union’s General Data Protection Regulation (EU GDPR) and the corresponding German privacy laws.

The original design of the observational study, informed consent form and study data including de-identified participant data sets are made available to researchers without any restriction. Access to data is unlimited after the date of publication.

**References in Supplementary Material**

1. Strupp M, Teufel J, Habs M, et al. Effects of acetyl-DL-leucine in patients with cerebellar ataxia: a case series. *J Neurol* 2013;260:2556–2561. doi: 10.1007/s00415-013-7016-x.

2. Kaya E, Smith DA, Smith C, et al. Beneficial Effects of Acetyl-DL-Leucine (ADLL) in a Mouse Model of Sandhoff Disease. *J Clin Med* 2020;9. doi: 10.3390/jcm9041050.

3. Bremova T, Malinová V, Amraoui Y, et al. Acetyl-dl-leucine in Niemann-Pick type C: A case series. *Neurology* 2015;85:1368–1375. doi: 10.1212/WNL.0000000000002041.

4. Kunz D, Stotz S, Bes F. Treatment of isolated REM sleep behavior disorder using melatonin as a chronobiotic. *J Pineal Res* 2021;71:e12759. doi: 10.1111/jpi.12759.

5. Sateia MJ. International classification of sleep disorders-third edition: highlights and modifications. *Chest* 2014;146:1387–1394. doi: 10.1378/chest.14-0970.

6. Oertel WH, Janzen A, Henrich MT, et al. Acetyl-DL-leucine in two individuals with REM sleep behavior disorder improves symptoms, reverses loss of striatal dopamine-transporter binding and stabilizes pathological metabolic brain pattern-case reports. *Nat Commun* 2024;15:7619. doi: 10.1038/s41467-024-51502-7.

7. Doppler K, Jentschke H-M, Schulmeyer L, et al. Dermal phospho-alpha-synuclein deposits confirm REM sleep behaviour disorder as prodromal Parkinson's disease. *Acta Neuropathol* 2017;133:535–545. doi: 10.1007/s00401-017-1684-z.

8. Yan S, Jiang C, Janzen A, et al. Neuronally Derived Extracellular Vesicle α-Synuclein as a Serum Biomarker for Individuals at Risk of Developing Parkinson Disease. *JAMA Neurol* 2024;81:59–68. doi: 10.1001/jamaneurol.2023.4398.

**Keywords**

Acetyl-Leucine, Parkinson’s Disease, Rapid-Eye-Movement-(REM-)Sleep Behavior Disorder, case report

**Glossary**

AL Acetyl-DL-Leucine

ALL Acetyl-L-Leucine

DAT SPECT Dopamine-Transporter Single-Photon Emission Computerized Tomography

iRBD isolated REM Sleep Behavior Disorder

PD Parkinson’s disease

PSG Polysomnography

RBD Rapid-Eye-Movement (REM) Sleep Behavior Disorder

RBD_SS RBD-severity sum-score

REM Rapid-Eye-Movement

UPSIT University of Pennsylvania Smell Identification Test

**Abstract**

**Objectives**

>50 % of Parkinson’s disease (PD) patients suffer from Rapid-Eye-Movement (REM)-sleep behavior disorder (RBD). We tested the effect of acetyl-leucine (AL) on RBD in PD.

**Method**

A 67-year-old man with the clinically (2016) and polysomnographically confirmed (2023) diagnosis of RBD was diagnosed with PD in 2019. In the same year he received levodopa/carbidopa plus rasagiline for PD-symptoms and melatonin for RBD-symptoms. Dopamine-transporter ligand binding (DAT)-SPECT was pathological. Smell identification test revealed hyposmia. In 06/2022 he started to additionally take AL 5 g/day. Patient and spouse recorded daily the RBD-severity (range 0-4) for 24 months. The “weekly RBD severity sum-score” (7d-RBD_SS; range 0-28) was calculated.

**Results**

His 7d-RBD_SS was 14 at baseline. After 4 weeks of AL-therapy it dropped below 6, after 9 weeks to 1 and varied the following 6 weeks between 1 and 4. In week 16, an AL-withdrawal was performed. The beneficial effect of AL was maintained until week 22. In week 23, RBD-symptoms reappeared (7d-RBD_SS: 11). AL-therapy was restarted. The 7d-RBD_SS further increased to 16 until week 26. At week 27, the 7d-RBD_SS fell to 2 and remained at this low level for the next 38 weeks. A second AL-washout showed a similar time course of the 7d-RBD_SS. Reintroducing AL-therapy reestablished the control of RBD symptoms until the end of the 2-year observation. AL was well tolerated.

**Discussion**

In this 2-year single case therapeutical study acetyl-leucine appears to markedly reduce the phenotype RBD in PD.
